# Longitudinal association of edentulism with cognitive impairment, sarcopenia and all-cause mortality among older Chinese adults

**DOI:** 10.1186/s12903-023-03015-w

**Published:** 2023-05-27

**Authors:** Yang Li, Chuan-Long Huang, Xiu-Zhen LU, Zi-Qing Tang, Yuan-Yin Wang, Ying Sun, Xin Chen

**Affiliations:** 1 Key Laboratory of Oral Diseases Research of Anhui Province, Stomatologic Hospital & College, Anhui Medical University, Hefei, China; 2 Department of Maternal, Child & Adolescent Health, School of Public Health, Anhui Medical University, 81th Meishan Road, Hefei, Anhui Province 230032 China

**Keywords:** Cognitive impairment, Edentulism, Longitudinal analysis, Mortality, Sarcopenia

## Abstract

**Background:**

Tooth loss may be a surrogate for systemic health and aging. However, no previous studies have systematically assessed multiple outcomes relevant to aging trajectory in this area, and many important confounders were not adjusted in most previous studies. This study aims to prospectively evaluate the associations of complete tooth loss (edentulism) with broad markers of sarcopenia, cognitive impairment and mortality.

**Methods:**

Data were derived from the China Health and Retirement Longitudinal Study, a nationally representative household study of the Chinese population aged 45 years and older. Multivariate Weibull proportional hazards regression was used to assess the association between edentulism with sarcopenia and all-cause mortality. Average changes in cognitive function by edentulism was estimated by mixed-effects linear regression models.

**Results:**

During the 5-year follow-up, the prevalence of edentulism among adults aged 45 and over was 15.4%. Participants with edentulism had a greater decline in cognitive function compared to those without (β=-0.70, 95%*CI*:-1.09, -0.31, *P* < 0.001). The association of edentulism and all-cause mortality for 45–64 age group (HR = 7.50, 95%*CI*: 1.99, 28.23, *P* = 0.003), but not statistically significant for the ≥ 65 age group (HR = 2.37, 95%*CI*: 0.97, 5.80, *P* = 0.057). Effects of edentulism on sarcopenia are statistically significant for all age groups (45–64 age group: HR = 2.15, 95%*CI*: 1.27, 3.66, *P* = 0.005; ≥65 age group: HR = 2.15, 95%*CI*: 1.27, 3.66, *P* = 0.002).

**Conclusions:**

These findings could have important clinical and public health implications, as tooth loss is a quick and reproducible measurement that could be used in clinical practice for identifying persons at risk of accelerated aging and shortened longevity, and who may benefit most from intervention if causality is established.

**Supplementary Information:**

The online version contains supplementary material available at 10.1186/s12903-023-03015-w.

## Background

China has one of the fastest growing ageing populations in the world. The latest results of China’s seventh population census show that in 2020, China’s population was 1.411 billion, of which 264 million were 60 years old and over, and 191 million were 65 years old and over, accounting for 18.7% and 13.5% of the total population respectively [1]. Comparing with the previous intercensal period, population aging in China accelerated in the last decade, which poses a major challenge to the health care system to ensure the health and longevity of an aging society. Oral health is an important intrinsic component of human health and well-being; however, oral health has been neglected in the current global health agenda. With the continuous progress of medical treatment and the massive investment of public health resources, most oral diseases can be prevented and cured, however, due to the irreversible aging and incomplete health protection measures, the elderly often do not receive necessary daily oral care, so it is impossible to maintain a good oral health level and oral hygiene status in old age. In addition to periodontal disease, there are a number of other oral diseases and functional problems that are common among the elderly, including edentulousness. Edentulism and severe tooth loss are still a major burden of oral disease disability worldwide [2]. Globally, about 30% of adults aged 65–74 have lost all of their teeth [3]. In view of the low utilization rate of oral health care for Chinese adults [4], it is speculated that the prevalence of edentulism in the elderly in China may be higher.

Friedman & Lamster [5] proposed that tooth loss may be a surrogate for systemic health and aging. Previous studies have reported lower rates of tooth loss among centenarians than younger members of the birth cohort aged 65–74 years [6]. Positive correlations between edentulism with cognitive impairment [7, 8], chronic illness [9, 10], depression [11], as well as cancer [12] are emerging in the literature. One recent research review reported that unrecovered patients with stage IV periodontitis showed lower cognitive status, higher drug intake and frailty [13]. Studies are needed to clarify the relationship between edentulism and tooth loss with the components of the frailty phenotype, e.g. low grip strength, slow gait speed and low physical activity [14].

Whilst reviewing the adverse effects of edentulism, no previous studies have systematically assessed multiple outcomes relevant to aging trajectory in this area, and many important confounders were not adjusted in most previous studies. Furthermore, primary evidence largely comes from high-income countries, whereas in low- and middle-income countries, this association has been underexplored. The present study seeks to prospectively evaluate the associations of complete tooth loss (edentulism) with broad markers of sarcopenia, cognitive impairment and mortality, in community-dwelling older adults participating in the China Health and Retirement Longitudinal Study (CHARLS).

## Methods

### Study population and design

**Fig. 1:**
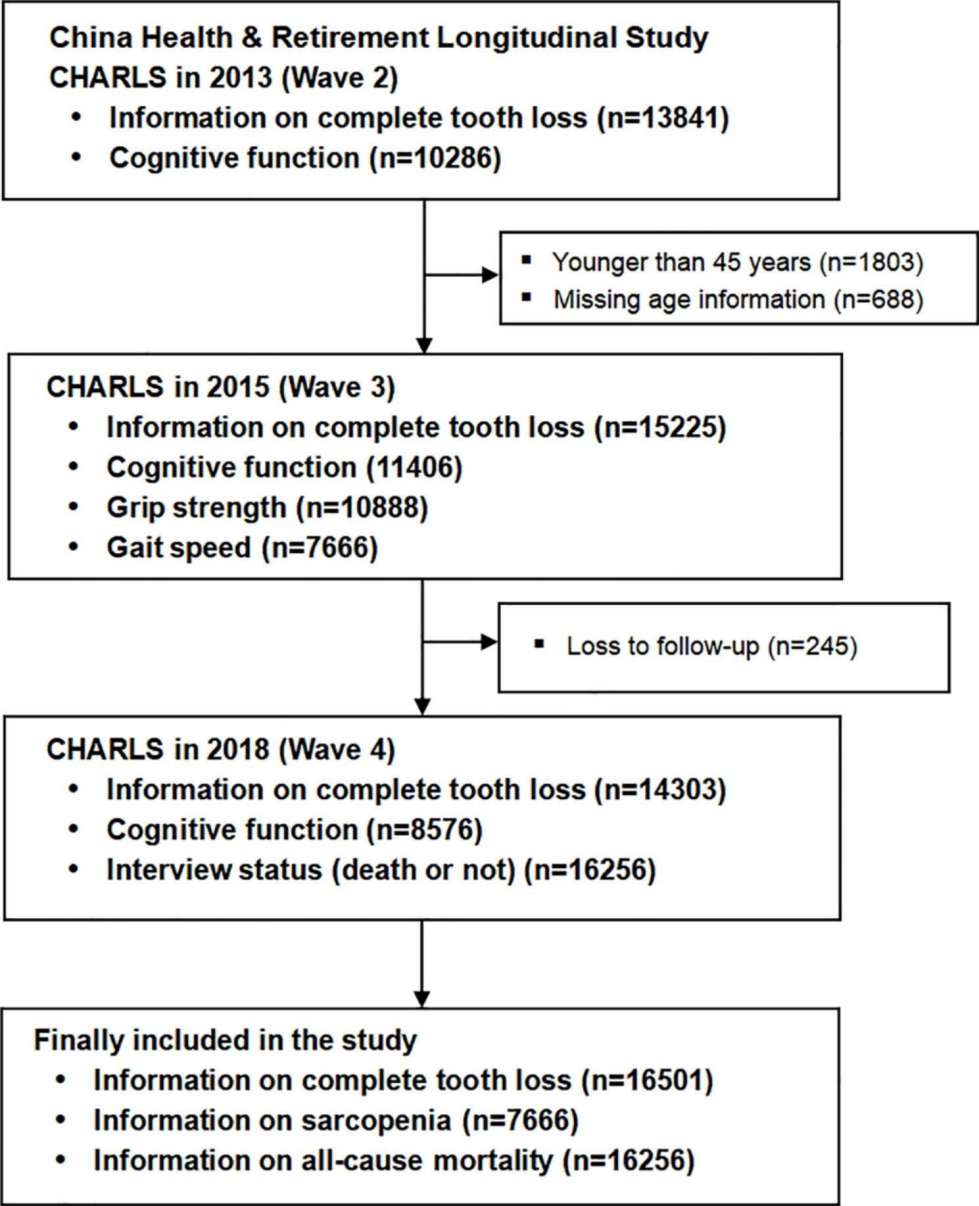
Study flowchart

This study analyzed a nationally representative data from the China Health and Retirement Longitudinal Study (CHARLS), which covered 450 villages or communities in 150 counties or districts of 28 provinces in China and was conducted among 45 years and older population. The baseline survey was conducted in 2011 (Wave 1, W1), through a four-stage, stratified, cluster probability sampling design. Considering the availability of complete tooth loss data, this study utilized data from the wave 2 in 2013 and two follow up surveys (wave 3, 2015; wave 4, 2018). Further details are provided elsewhere [15]. The exclusion criteria were as follows: (i) those individuals who did not report an edentulous status; (ii) age was less than 45 years old; and (iii) those with missing data. The CHARLS study was approved by the Biomedical Ethics Review Committee of Peking University (approval number IRB00001052–11,015). All interviewees provided informed consent. The research has been performed in accordance with the Declaration of Helsinki. The data is available freely at http://charls.pku.edu.cn/. This study included 19,157 participants with information on complete tooth loss at Wave 2 (Fig. 1).

### Measurements

#### Edentulism

Information on complete tooth loss was assessed in Wave 2 to Wave 4, through one question: “Have you lost all of your tooth?”, the variable had two options (yes or no).

#### Cognitive function

Cognitive function was measured from two dimensions [16], the first dimension is episodic memory divided into immediate word recall (0–10 point) and delayed word recall (0–10 point), asking respondents to repeat, regardless of the order, 10 Chinese nouns immediately and 5 min later. The second dimension was mental status based on questions of the Telephone Interview of Cognitive Status (TICS) battery, which established to understand the integrity or mental state of the individual [17]. Mental status was measured from orientation, visuo-construction, and attention. CHARLS examined orientation through the respondents’ recognition of date (month, day, year), and the day of the week (0–4 points). Visuoconstruction (0–1 point) was assessed by asking individuals to exactly redraw a picture of overlapping pentagons on the paper. Attention (0–5 points) was measured by serial subtraction of 7 from 100 for five times. The total cognitive function score varied from 0 to 30, with higher values meaning better cognitive function [18].

#### Assessment of sarcopenia

According to the AWGS criteria, sarcopenia was diagnosed if participants had low muscle mass plus low muscle strength or low physical performance [19].

##### Measurement of muscle strength

AWGS 2019 recommends using handgrip strength to indicate skeletal muscle strength. Handgrip strength (kg) was measured in the dominant hand and non-dominant hand, with the participant squeezing a YuejianTM WL-1000 dynamometer (Nantong Yuejian Physical Measure-ment Instrument Co., Ltd., Nantong, China) as hard as possible. According to the suggestion of AWGS 2019, the cut-off points for low handgrip strength are < 18 kg in women and < 28 kg in men.

##### Measurement of muscle mass

The muscle mass was estimated by the appendicular skeletal muscle mass (ASM) using a previously validated anthropometric equation in a Chinese population [20]:

1 $$\displaylines{{\text{0}}{\text{.193*weight + 0}}{\text{.107*height - 4}}{\text{.156*sex1 - }} \cr {\text{0}}.{\text{037*death}}\_{\text{age - 2}}{\text{.631}} \cr}$$

The height, body weight and age were measured in centimeters, kilograms and years, respectively. For sex, the value 1 represented men, and the value 2 represented women. The agreement of the ASM equation model and dual X-ray absorptiometry (DXA) was strong. After calculating the ASM, the height-adjusted muscle mass (ASM/Ht^2^) was calculated using the ASM divided by the square of the height in meters. The ASM/Ht^2^ values of < 5.63 kg/m^2^ in women and < 7.05 kg/m^2^ in men were considered as low muscle mass. In terms of physical performance, the gait speed and the chair stand test were performed using the method described by Wu et al. [21]0.10 Further details about the definitions for sarcopenia components in the CHARLS have been described previously.10 According to AWGS 2019, the criteria for low physical performance are 6-m walk < 1.0 m/s, or 5-time chair stand test ≥ 12 s [19].

#### All-cause mortality

The death information in CHARLS was determined by the exit interview status (alive or dead) of participants in Waves 3 and Wave 4. Considering the exact time of death was not recorded, the median of the two follow-up times was used as the survival time.

#### Covariates

We selected covariates that may confound the relationship based on a review of literature. The selected covariates should have an impact on the physical fragility, cognitive impairment, and overall mortality and been collected in the CHARLS. Because the sample size of this study is large and statistical power is strong, we included as many covariates as possible to minimize the potential confounding effect. The covariates for our analyses included sociodemographic characteristics (age, sex, household income, education, occupation status, marital status and residence), routine physical checkup (weigh, height, and blood pressure), lifestyle behaviors (smoking, alcohol drinking and sleep duration), self-reported chronic conditions (hypertension, diabetes/hyperglycaemia, cancer, lung disease, stroke, heart disease, psychological disease, arthritis, liver disease, kidney disease, digestive disease, asthma and memory-related disease). Details on the precision of the covariates are provided in the appendix.

### Statistical analyses

Baseline characteristics of participant with and without edentulism were compared using *χ*^2^ tests for categorical measures and *t* tests for continuous measures. Statistical analysis was performed from April to August, 2022. Average changes in cognitive function over Wave 2 to Wave 4 by edentulism status was estimated using mixed-effects linear regression models. We ran three incremental models: Model 1 was adjusted for sociodemographic factors (age, sex, household annual income and education); Model 2 was additionally adjusted for health-related factors including smoking, drinking, activities of daily living, sleep duration and body mass index; and Model 3 was additionally adjusted for self-reported medical history (hypertension, diabetes, respiratory illnesses, stroke and heart disease and other chronic illnesses at Wave 4). Sensitivity analysis was conducted in subgroups of population without stroke or heart diseases to examine the stability of results.

Multivariate Weibull proportional hazards regression model before and after adjusting for confounding factors (sociodemographic factors, health-related factors, and self-reported medical history) were used to estimate the associations between edentulism with all-cause mortality and sarcopenia. Hazard ratios (HRs) and 95% CIs were reported for both unadjusted and covariate-adjusted models. Statistical significance was set at two-sided *P* < 0.05. Analyses were performed using STATA v15 statistical software.

## Results

The characteristics of study participants according to edentulous groups are presented in Table 1. The mean age of the 16 501 participants was 58.8 (standard deviation [SD], 9.5) years, and 51.2% were women. Nearly 2/3 (66.4%) of participants received primary education or less.

The prevalence of edentulism among adults aged 45 and over was 15.4% (2537/16,501), with 10.1% for age group 45–64 years, 25.6% for age group 65–74 years and 44.2% for age group 75 and over. Compared to those without edentulism, edentulous participants were older, being female, less educated, and had lower BMI, lower household income, and lower alcohol use (all *P*s < 0.001).

As shown in Table 1, compared to those without edentulism, the prevalence of high blood pressure at 2013 wave was significantly higher among participants with edentulism (*P* < 0.001). The prevalence of heart diseases was similar at 2013 between the two groups (14.1% vs. 13.2%, *P* = 0.257), but significantly increased at 2015 (20.2% vs. 17.6%, *P* = 0.004) and 2018 (25.1% vs. 21.1%, *P* < 0.001) among elder population with edentulism.

Cognitive function score declined with age during the follow-up period in all participants (Table 2). Participants with edentulism had a greater decline compared to those without. The associations remained significant after adjusted prevalent cardiovascular diseases, e.g. high blood pressure, diabetes, stroke and heart disease at Wave 4 (*β*=-0.70, 95%*CI*:-1.09, -0.31, *P* < 0.001). Results from sensitivity analyses excluding participants with baseline cardiovascular diseases were qualitatively similar (*β*=-0.68, 95%*CI*: -1.10, -0.26, *P* = 0.001).

**Table 1:** Characteristics of study participants by edentulism at 2013 in China Health and Retirement Longitudinal Study

Characteristics	Total sample		Edentulism during 2013–2018 (n = 16,501)		*P*-values
			Yes (n = 2537)	No (n = 13,964)	
**Age at 2013 (years), mean (SD)**	16,501		65.5 ± 9.9	57.6 ± 8.8	< 0.001
45–64 years	12,271		1244 (10.1)	11,027 (89.9)	< 0.001
65–74 years	3104		795 (25.6)	2309 (74.4)	
>=75 years	1126		498 (44.2)	628 (55.8)	
**Female, n (%)**					0.001
Female	8449		1374 (16.3)	7075 (83.7)	
Male	8052		1163 (14.4)	6889 (85.6)	
**Education at 2013, n (%)**	14,403				< 0.001
Primary or less	9570		1781 (18.6)	7789 (81.4)	
Lower secondary	3123		314 (10.1)	2809 (89.9)	
Upper secondary & vocational	1500		99 (6.6)	1401 (93.4)	
Tertiary	210		16 (7.6)	194 (92.4)	
**Household income at 2013, mean (SD)**	9377		10376.7 ± 12498.0	18941.3 ± 24766.4	< 0.001
**BMI at 2013, mean (SD)**	10,145		23.1 ± 5.2	24.1 ± 3.7	< 0.001
**Current smoke at 2013, n (%)**	13,943		520 (25.4)	3215 (27.0)	0.145
**Alcohol use at 2013, n (%)**	13,943		565 (27.6)	4055 (34.1)	< 0.001
**High blood pressure at 2013, n (%)**	13,277		580 (26.4)	2434 (22.0)	< 0.001
**Dentures, n(%)**	14,303		516 (61.8)	4020 (29.8)	< 0.001
**Stroke, n (%)**					
W2 (2013)	13,253		72 (3.3)	304 (2.8)	0.181
W3 (2015)	13,810		95 (4.5)	450 (3.8)	0.150
W4 (2018)	13,963		192 (9.4)	932 (7.8)	0.019
**Heart diseases, n (%)**					
W2 (2013)	13,218		310 (14.1)	1451 (13.2)	0.257
W3 (2015)	13,719		425 (20.2)	2044 (17.6)	0.004
W4 (2018)	13,961		514 (25.1)	2516 (21.1)	< 0.001
**Global cognition score, mean (SD)**					
W2 (2013)	10,286		13.9 ± 4.9	15.5 ± 4.6	< 0.001
W3 (2015)	11,294		13.1 ± 4.8	14.9 ± 4.7	< 0.001
W4 (2018)	8575		13.8 ± 5.2	15.7 ± 5.0	< 0.001
**Sarcopenia at 2015, n (%)**	7666		131 (11.3)	242 (3.7)	< 0.001
**Death at 2018, n (%)**	16,256		217 (9.5)	496 (3.6)	< 0.001

**Table 2:** Average changes in cognitive function during 2013 to 2018 waves by edentulism in CHARLS using mixed-effects linear regression models

Variables	*β*-coefficient (95% confidence) Interval), cognitive function during Wave 2 to Wave 4		
	Model 1 ^a^	Model 2 ^a^	Model 3 ^a^
Total sample			
**Edentulism**			
No	Reference	Reference	Reference
Yes	-0.50 (-68, -0.33) ^**¶**^	-0.75 (-1.13, -0.38) ^**¶**^	-0.70 (-1.09, -0.31) ^**¶**^
**Age**	-0.11 (-0.12, -0.10) ^**¶**^	-0.12 (-0.14, -0.11) ^**¶**^	-0.12 (-0.14, -0.11) ^**¶**^
**Female**	-0.53 (-0.65, 0.42) ^**¶**^	-1.02 (-1.31, -0.74) ^**¶**^	-1.00 (-1.31, -0.70) ^**¶**^
**Education**			
Primary or less	Reference	Reference	Reference
Lower secondary	2.92 (2.78, 3.06) ^**¶**^	3.04 (2.74, 3.35) ^**¶**^	3.15 (2.84, 3.47) ^**¶**^
Upper secondary	4.41 (4.23, 4.59) ^**¶**^	4.50 (4.05, 4.94) ^**¶**^	4.64 (4.17, 5.11) ^**¶**^
Tertiary	5.79 (5.38, 6.21) ^**¶**^	6.27 (4.99, 7.54) ^**¶**^	6.31 (4.89, 7.73) ^**¶**^
**Participants without stroke or heart diseases at Wave 2**			
**Edentulism**			
No	Reference	Reference	Reference
Yes	-0.56 (-0.89, -0.23) ^**§**^	-0.70 (-1.12, -0.28) ^**§**^	-0.68 (-1.10, -0.26) ^**§**^

^**a**^ Model 1 was adjusted for age, sex, household annual income and education; Model 2 was further adjusted for smoking, alcohol drinking, sleep duration, body mass index and dentures; in Model 3, hypertension, diabetes, respiratory illnesses, stroke and heart disease and other chronic illnesses at Wave 4 were added

Table 3 shows the associations between edentulism with mortality and sarcopenia from the random-intercept Weibull hazard models, respectively. The mortality incidence rate among older adults with edentulism was 19.9 per 1000 person-years (95%*CI*: 17.4, 22.7), compared to 8.1 per 1000 person-years (95%*CI*: 7.4, 8.8) among control group. Similarly, the incidence of sarcopenia during 2013 to 2015 waves was significantly higher in edentulous participants (28.3 cases per 1000 person years, 95%*CI*: 22.8, 35.1) compared to their counterparts from control group (10.4 cases per 1000 person years, 95%*CI*: 9.3, 11.7).

**Table 3:** Association of edentulism with mortality and sarcopenia estimated by random-intercept Weibull hazard models

		Cases	Incidence Rate (95%CI), per 1000 Person-Years		Hazard Ratio / Odds Ratio ^a^			
					Model 1		Model 2	
**Incidence of death at Wave 4**								
**Total**								
No edentulism group		496	8.1 (7.4, 8.8)		Reference		Reference	
Edentulism group		217	19.9 (17.4, 22.7)		2.52 (2.15, 2.95) ^**¶**^		2.88 (1.39, 5.98) ^**§**^	
**Female**		502	11.8 (10.8, 12.9)				0.32 (0.12, 0.81) *	
**Education**								
Primary or less		687	12.8 (11.9, 13.8)		-		Reference	
Lower secondary		127	7.4 (6.2, 8.8)		-		0.51 (0.15, 1,74)	
Upper secondary and higher		52	5.5 (4.2, 7.2)		-		0.80 (0.17, 3.69)	
**Aged 45–64**								
No edentulism group		199	4.2 (3.6, 4.8)		Reference		Reference	
Edentulism group		34	6.2 (4.4, 8.6)		2.52 (2.15, 2.95) ^**¶**^		7.50 (1.99, 28.23) ^**§**^	
**Aged 65 or older**								
No edentulism group		297	19.4 (3.6, 24.4)		Reference		Reference	
Edentulism group		183	33.8 (29.3, 39.1)		1.59 (1.32, 1.91) ^**¶**^		2.37 (0.97, 5.80)	
**Incidence of sarcopenia at Wave 3**								
**Total**								
No edentulism group		291	10.4 (9.3, 11.7)		Reference		Reference	
Edentulism group		82	28.3 (22.8, 35.1)		3.37 (2.72, 4.17) ^**¶**^		1.70 (1.32, 2.20) ^**¶**^	
**Female**		287	16.7 (14.8, 18.7)				1.04 (0.77, 1.42)	
**Education**								
Primary or less		442	21.2 (19.3, 23.2)		-		Reference	
Lower secondary		37	5.6 (4.0, 7.7)		-		0.43 (0.25, 0.75) ^**§**^	
Upper secondary and higher		14	3.9 (2.3, 6.6)		-		0.46 (0.21, 1.04)	
**Aged 45–64**								
No edentulism group		74	3.9 (3.1, 4.9)		Reference		Reference	
Edentulism group		23	10.6 (7.1, 16.0)		3.22 (2.01, 5.16) ^**¶**^		2.15 (1.27, 3.66) ^**§**^	
**Aged 65 or older**								
No edentulism group		168	30.3 (26.1, 35.2)		Reference		Reference	
Edentulism group		108	49.8 (41.2, 60.1)		1.65 (1.30, 2.11) ^**¶**^		1.58 (1.19, 2.09) ^**§**^	

^**a**^ Model 1 was unadjusted for covariates; Model 2 was adjusted for age, sex, education, household annual income, smoking, alcohol drinking, sleep duration, dentures, body mass index, hypertension, diabetes, respiratory illnesses, stroke and heart disease and other chronic illnesses at Wave 4

**Fig. 2:**
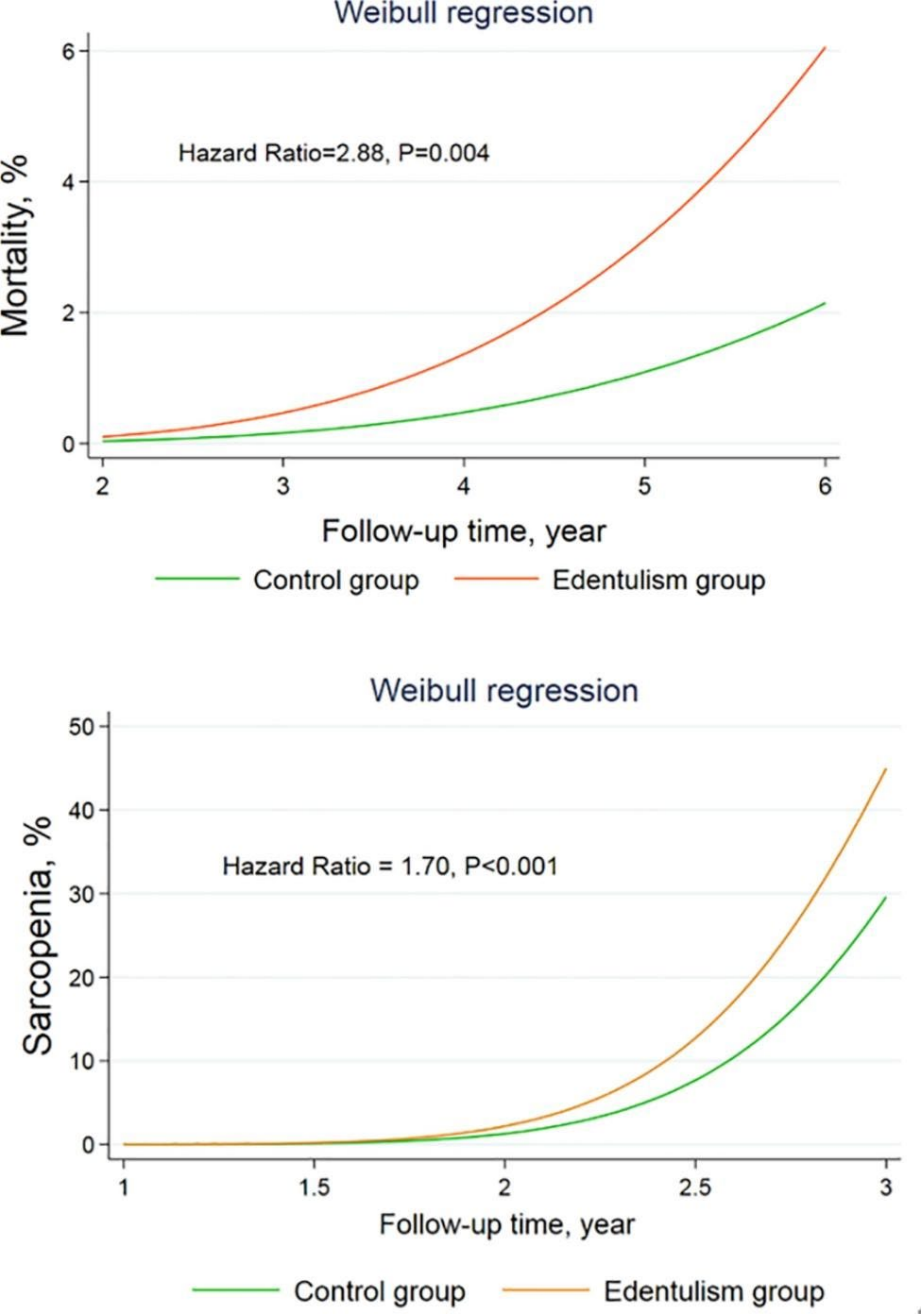
Weibull random-intercept survival regression models for survival and sarcopenia by edentulism groups during Wave 2 to Wave 4

We calculated two regression models—the null model is an empty model without any covariates, and the full model includes all covariate—gender, educational level, BMI, baseline cardiovascular diseases, diabetes, respiratory illnesses and other chronic morbidities, physical activity, current smoking and drinking behavior, and household income. In both models, the variation in mortality and sarcopenia risk was statistically significant. The median hazard of mortality and sarcopenia is 2.88- and 1.70-times higher among edentulous participants, compared with control groups (mortality: 95%*CI*: 1.39, 5.98, *P* = 0.004; sarcopenia: 95%*CI*: 1.32, 2.20, *P* < 0.001) (Table 3; Fig. 2).

We age-stratify our analysis by separately examining each of the two age groups, 45–64 years and 65 years or older. The results confirm the association of edentulism and all-cause mortality for 45–64 age group (HR = 7.50, 95%*CI*: 1.99, 28.23, *P* = 0.003), but not statistically significant for the ≥ 65 age group (HR = 2.37, 95%*CI*: 0.97, 5.80, *P* = 0.057). Effects of edentulism on sarcopenia are statistically significant for all age groups (45–64 age group: HR = 2.15, 95%*CI*: 1.27, 3.66, *P* = 0.005; ≥65 age group: HR = 2.15, 95%*CI*: 1.27, 3.66, *P* = 0.002).

## Discussion

Based on the nationally representative CHARLS cohort, we found that edentulism was related to subsequent cognitive impairment, risk for sarcopenia and all-cause mortality among 16,501 elderly population in China. Moreover, all the association between complete tooth loss and these aging relevant outcomes remained highly significant in the competing risk model with fully multivariable analysis. As far as we know, this is the primary prospective study to assess cumulative edentulism while studying associations with subsequent cognitive impairment, risk of sarcopenia and all-cause mortality.

Using a prospective design, our results show robust evidence of an association of lower education and household income with incident edentulism among Chinese adults aged 45 and above. These findings were consistent with previous studies [22, 23], and highlighted the importance of addressing “upstream” determinants of oral health, especially for the most vulnerable populations in order to ensure compliance with the principle of equity [24].

A growing number of epidemiological studies focused on the effect of oral health on cognitive function [25, 26], however, less studies paid attention to edentulism and with mixed findings [8, 21, 27]. The inconformity may be attributed to differences in the age range and ethnicity of the subjects, as well as differences in the measures and methods of cognitive function and oral health. Our findings are consistent with Lu et al. [8], demonstrating that the relationship between edentulism and lower level of episodic memory and psychological integrity at follow-up among Chinese elder population.

Certain physiological mechanisms could link edentulism to poor cognitive function. A recent animal study observed that tooth loss in the juvenile Sprague–Dawley rats will reduce the number of pyramidal neurons in the hippocampus, inhibit the expression of BDNF, TrkB, AKT1, and NR2B, and eventually lead to cognitive dysfunction [28]. Other potential explanations include the long-term inflammatory stress pathway, which involves metabolic disorders, microbial-gut-brain axis, the activation of microglia and astrocytes, and inflammatory cascade effect in central nervous system [29]. Thus, our study generated important scientific evidence for early detection of cognitive impairment and dementia. Finally, the non-significant associations in the middle-aged group may have resulted from the fact that this group was still relatively young and might not have experienced a significant decline in cognitive function during the 5-year study period.

Several previous studies have evaluated whether edentulism and partial edentulism may be predictive of shortened longevity [30–34], mostly conducted in high- and upper-middle-income countries, which revealed small association. Our study has further extended such associations, indicating that late middle-aged adults (45–64 yeas) with edentulism presented a substantially (7.5-times) higher risk for all-cause mortality than the reference population, while edentulous elder population over 65 years of age demonstrated no extra risk of mortality. In contrast, a retrospective study among 1385 elderly individuals > 75 years old from Shanghai, China found tooth loss was the potential predictors of mortality [35]. Compared to elder population, total tooth loss at a younger age is more likely to represent cumulative exposure to adverse oral and overall health status, and/or lifetime stressors [36], where the effect of edentulism on mortality is maximized. Our findings suggest that the provision of routine dental care and other dental public health interventions might lead to reductions in early death.

Our findings demonstrated a consistent association between edentulism and sarcopenia in both community-dwelling middle-aged and older populations, suggesting that the association between edentulism with declining muscle strength and muscle mass over time is robust. However, there is emerging evidence that tooth loss is associated with grip strength and sarcopenia, and most of these studies are observational and cross-sectional [37–39]. The predictive value of tooth loss on future sarcopenia in older adults are still unknown. Our findings help address this gap illustrating that complete edentulism independently predicts declining grip strength and muscle mass over time among participants aged 45 years or older. Our findings suggest that, in addition to health factors, oral health indicators are important considerations in determining sarcopenia risk status and prevention strategies in the elderly population.

The present study found that the self-reported edentulism in adults aged 45 and above is 15.4%, which is higher than previous studies [12, 39]. According to the World Health Organization Study on Global AGEing and Adult Health (SAGE), the prevalence of edentulism in Chinese adults aged 50 years and over is 8.9% [23]. Data from the 4th National Oral Health Epidemiology Survey in China found the prevalence of edentulism aged 65–74 years was 4.42%. According to the study of Khazaei et al. [40]. The rate of edentulism in developed countries shows a steady downward trend [41, 42], but in developing countries, the situation is quite different. Professional dental care services are not widespread in China, and many dental treatment programs are not covered by medicare and are often unaffordable for many, posing a serious public health challenge for policymakers. Our study will provide a novel perspective for the formulation of public health policy. The results of the present study suggest that epidemiological studies of edentulous loss and its potential later burden will be of great significance for clinical treatment planning and later strategy development, as well as for the assessment of public health needs and service planning. The data will also have an impact on practical actions that include oral health to stimulate systemically health and achieve the major goals of oral health in society [43].

There are also limitations to our research. First of all, because what we are analyzing is secondary data, we can only choose indicators from the CHARLES questionnaire. Self-reported edentulous loss may be affected by recall bias. In addition, the queue records the minimum data related to the use of dentures, so we cannot investigate the potential modification effects of the observed associations. Collecting information related to oral health status progression and other related health outcomes, such as dentition defects, periodontal disease, use of dentures, oral health history, and masticatory function, should be the focus of future edentulous research. Second, the evaluation of edentulism, cognitive decline and mortality in this study was followed up for 6 years. Thus, additional follow-up is necessary to better examine the associations between edentulism and multiple health outcomes. Third, the data for most chronic conditions were self-reported and the prevalence of some chronic conditions were low. Therefore, there might have been insufficient power to detect a significant difference. Fourth, considering the exact time of death was not recorded, the incidence of mortality may be undermined by absence of precise time of death. Finally, despite the more comprehensive approach to addressing issues of confounding compared with prior studies, residual confounding from unknown or unmeasured factors still remains possible.

## Conclusions

In conclusion, in this large nationwide cohort study representing the Chinese mid-adulthood and older population, edentulism is associated with a higher risk of cognitive decline, sarcopenia and all-cause mortality, independent of a wide range of sociodemographic, health, and behavioural confounders. These findings could have important clinical and public health implications, as tooth loss is a quick and reproducible measurement that could be used in clinical practice for identifying persons at risk of accelerated aging and shortened longevity, and who may benefit most from intervention if causality is established. Public policy and health-care system strategies aimed at oral health promotion and disease prevention, as well as increasing access to oral health services for older adults, are important overarching goals that will improve tooth retention, thereby decreasing disability and potentially increasing longevity.

## Electronic supplementary material

Below is the link to the electronic supplementary material.

Supplementary Material 1

## Data Availability

Please contact China Health and Retirement Longitudinal Study (CHARLS) for data requests at http://charls.pku.edu.cn/.

## Abbreviations

CHARLS: China Health and Retirement Longitudinal Study
TICS: Telephone Interview of Cognitive Status
AWGS: Asian Working Group for Sarcopenia
ASM: Appendicular Skeletal Muscle Mass
DXA: Dual X-ray Absorptiometry
HR: Hazard ratio
CI: Confidence interval
SD: Standard Deviation
BMI: Body Mass Index
BDNF: Brain-derived Neurotrophic Factor
TrkB: Tyrosine Kinase receptor B
AKT1: v-akt murine thymoma viral oncogene homolog 1
NR2B: N-methyl-D-aspartate receptor subunit 2B
SAGE: Study on Global AGEing and Adult Health.
