# Supplementary material for: Longitudinal association of edentulism with cognitive impairment, sarcopenia and all-cause mortality among older Chinese adults
Source: BMC Oral Health. 2023 May 27;23:333. doi: 10.1186/s12903-023-03015-w (PMC10225090; doi:10.1186/s12903-023-03015-w)
Supplement: Supplementary file 1 — Supplementary Material 1 [file 12903_2023_3015_MOESM1_ESM.docx]

**Covariates**

CHARLS collects self-reported information for specific chronic conditions by asking participants “Have you ever been told by a doctor that you have the specific condition?”. Chronic diseases were all dichotomized as yes versus no, which included hypertension, high blood sugar/diabetes, cancer, chronic lung disease, stroke, heart diseases, arthritis, dyslipidemia, liver disease, kidney disease, digestive disease, asthma, memory-related disease and emotional, nervous or psychiatric disorders. Besides the positive answer, hypertension and diabetes/hyperglycaemia can also be separately diagnosed by [systolic blood pressure](https://www.sciencedirect.com/topics/medicine-and-dentistry/systolic-blood-pressure) ≥ 140 mmHg and/or [diastolic blood pressure](https://www.sciencedirect.com/topics/medicine-and-dentistry/diastolic-blood-pressure) ≥ 90 mmHg and fasting [blood glucose](https://www.sciencedirect.com/topics/biochemistry-genetics-and-molecular-biology/glucose-blood-level) ≥ 6.1 mmol/L and/or postprandial 2 h blood glucose ≥ 7.8 mmol/L. Blood pressure levels were recorded three times separately at 45-s intervals by trained reviewers through using HEM-7200 electronic monitor (Omron, Dalian, Japan). In addition, sensitivity analysis was conducted for objective measures or self-reported information to define hypertension and diabetes/hyperglycaemia, respectively.

The demographic characteristics in the present study included age, gender, smoking, drinking, body mass index (BMI), educational level, marital status, occupation status, and residence. Education status was dichotomized as illiterate versus literate. Income level was classified into above-median household income or below-median household income. Occupation status was categorized as agriculture work and below (including agriculture work, unemployed, never work and retired without pension) and non-agriculture work (including non-agricultural work for wages, non-agricultural self-employed work, non-agricultural work without pay for a family business and retired with pension). Smoking status was dichotomized as ever versus never, and drinking status as ever versus never. BMI was calculated through weight (kg) divided by height squared (m^2^). Marital status was classified either as married or cohabiting, or living alone.
